# Supplementary material for: Premise plumbing bacterial communities in four European cities and their association with Legionella
Source: Front Microbiomes. 2023 Jun 19;2:1170824. doi: 10.3389/frmbi.2023.1170824 (PMC12993568; doi:10.3389/frmbi.2023.1170824)

Supplementary Material

**Premise plumbing bacterial communities in four European cities and their association with *Legionella***

Maria Scaturro^1,9^, Federica Del Chierico^2^, Yair Motro^3^, Angeliki Chaldoupi^4^, Anastasia Flountzi^4,9^, Jacob Moran-Gilad^3,9^, Antonietta Girolamo^1^, Thomai Koutsiomani^4^, Bozena Krogulska^5^, Diane Lindsay ^6,9^, Renata Matuszewska^5^, Georgios Papageorgiou^4^, Katarzyna Pancer^5^, Nikolaos Panoussis^4^, Maria Cristina Rota^1^, Søren Anker Uldum^7,9^, Emmanuel Velonakis^8^, Dominique L. Chaput^6^$\dagger$, Maria Luisa Ricci^1,9^$\dagger$,

$\dagger$ Both authors contributed equally to the work. To whom correspondence should be addressed: [dominique.chaput@ggc.scot.nhs.uk](mailto:dominique.chaput@ggc.scot.nhs.uk), [marialuisa.ricci@iss.it](mailto:marialuisa.ricci@iss.it)

1. National Reference Laboratory for Legionella, Istituto Superiore di Sanità, Department of Infectious Disease, Rome, Italy

2. Multimodal Laboratory Medicine Research Area, Bambino Gesù Children’s Hospital, IRCCS,Unit of Human Microbiome, Rome, Italy

3. Department of Health Systems Management, School of Public Health, Faculty of Health Sciences, Ben Gurion University of the Negev, Beer Sheva, Israel

4. Central Public Health Laboratory, Hellenic National Public Health Organization, Vari-Athens, Attiki, Greece

5. Department of Virology, National Institute of Public Health, NIH (NIPH-NIH), Warsaw, Poland

6. Scottish Microbiology Reference Laboratories, Glasgow, Scotland

7. Staten Serum Institute, Department of Bacteria, Parasites and Fungi, Copenhagen S, Denmark

8. Hellenic National School of Public Health, Athens, Greece

^9^ESCMID Study Group for Legionella Infections (ESGLI), 4001 Basel, Switzerland.

## Supplementary Figures


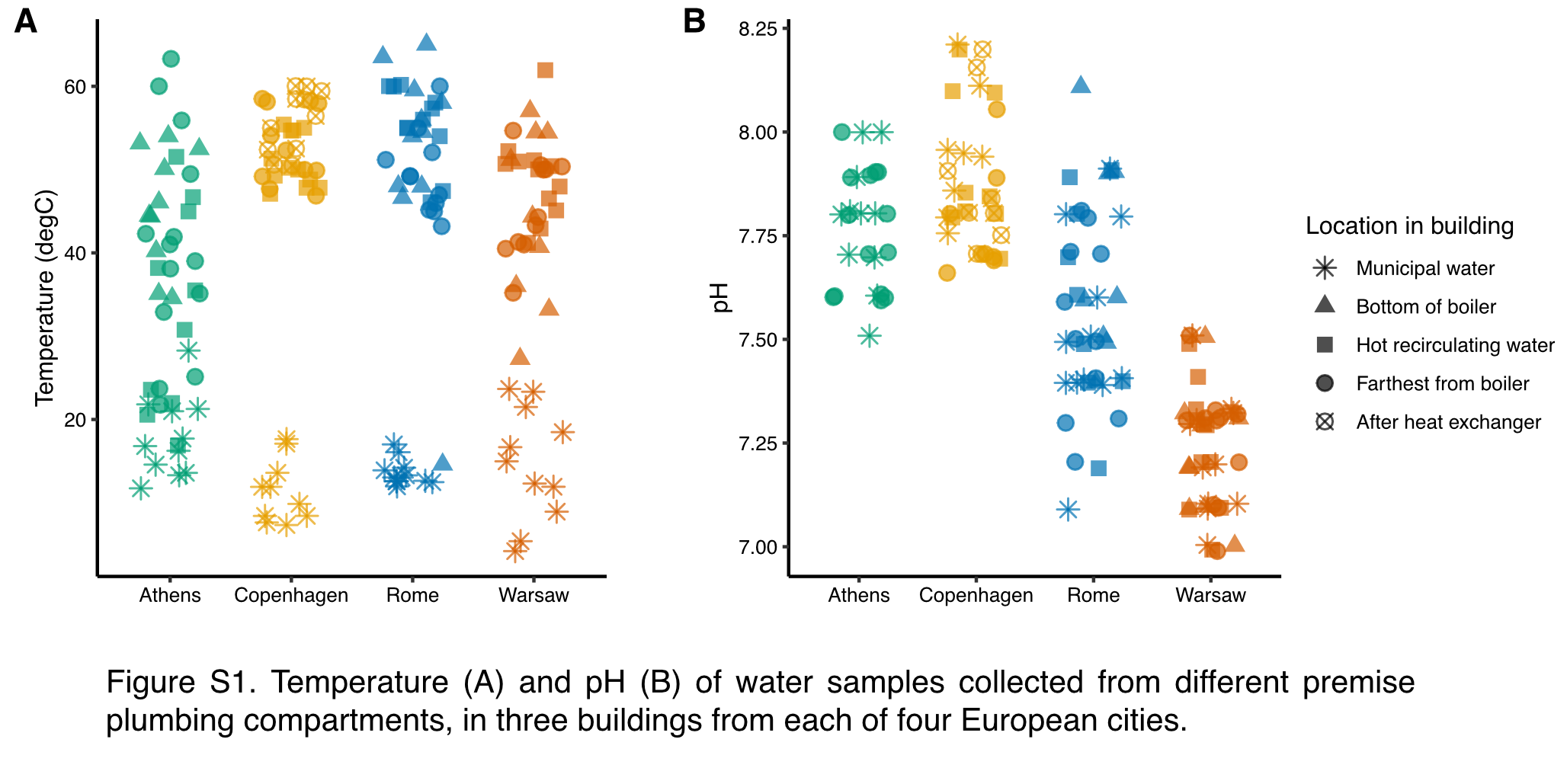


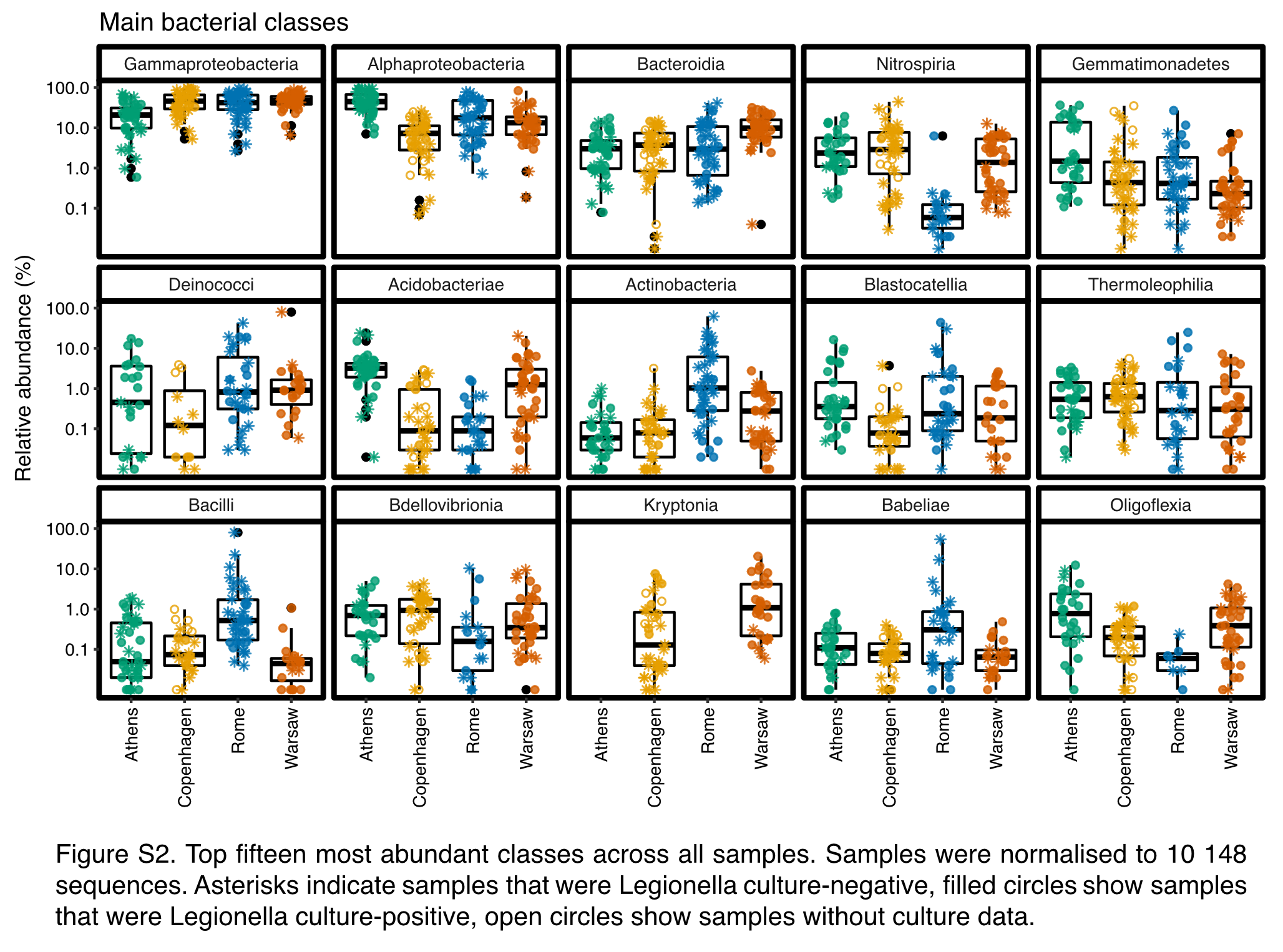


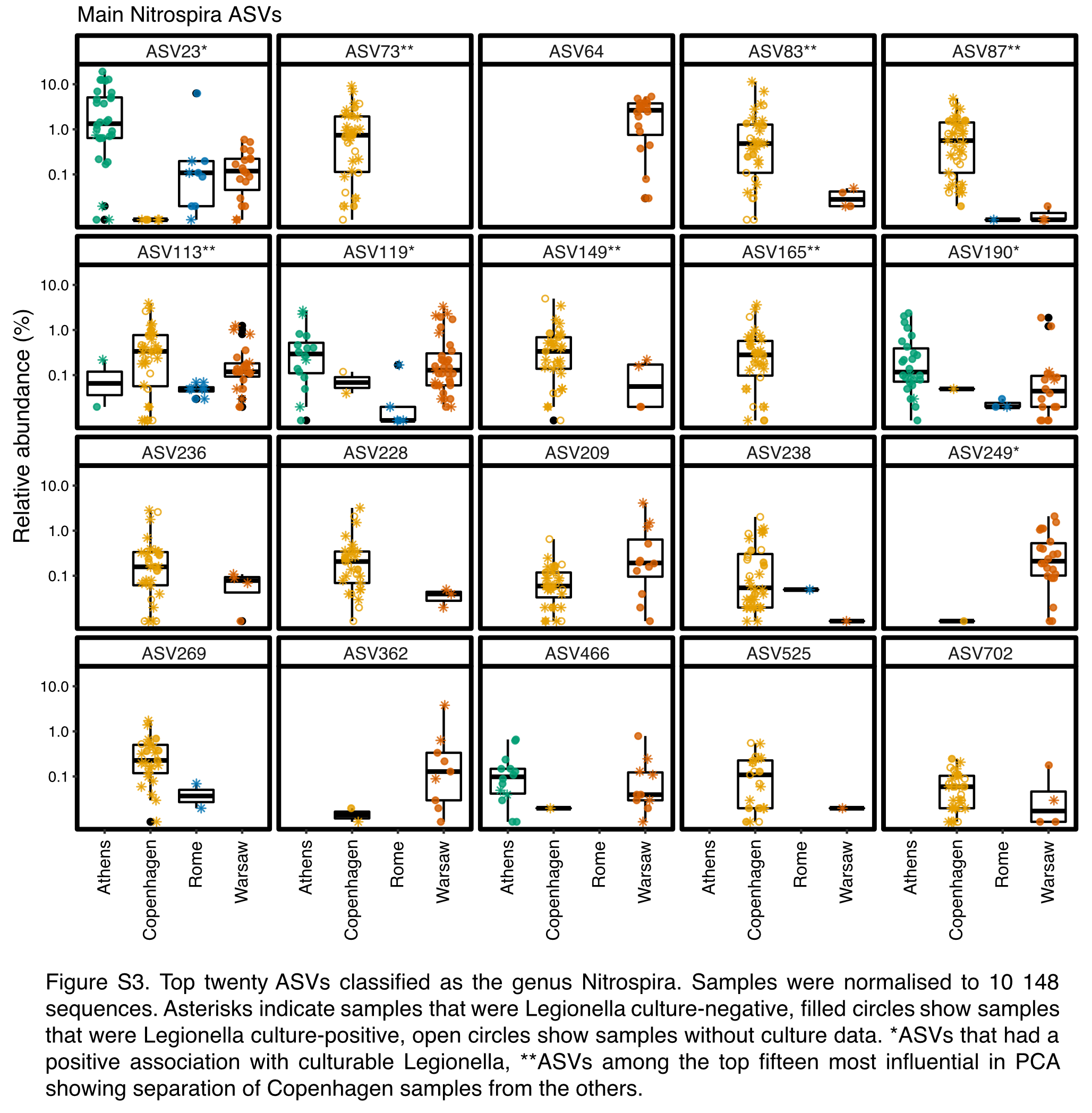

Supplement: Supplementary file 1 [file DataSheet_1.docx]
