# Supplementary material for: Premise plumbing bacterial communities in four European cities and their association with Legionella
Source: Front Microbiomes. 2023 Jun 19;2:1170824. doi: 10.3389/frmbi.2023.1170824 (PMC12993568; doi:10.3389/frmbi.2023.1170824)
Supplement: Supplementary file 2 [file Table_1.pdf]

Supplemental data

Statistical tables

ASV population richness estimates

Estimated population richness was calculated using `breakaway` (version 4.7.5). Mixed effects model was run with `betta_random`, which takes into account the uncertainty around the population richness estimate. City and presence/absence of culturable *Legionella* were specified as fixed effects (including the interaction), and building compartment ID was specified as a random effect (`betta_random` accepts a single random effect term).

*Table S1. Population richness: Output of mixed effect model run with the breakaway package, which accounts for the uncertainty around the richness estimates. Model formula: Estimated.richness ~ City * CFU_pres_abs + (1 | Bldg_loc_ID)*

	Estimates	Standard Errors	p-values
(Intercept)	97.498	11.997	0.000
CityCopenhagen	449.793	42.239	0.000
CityRome	43.083	20.113	0.032
CityWarsaw	490.092	26.931	0.000
CFU_pres_abspos	122.030	17.478	0.000
CityCopenhagen:CFU_pres_abspos	148.919	124.479	0.232
CityRome:CFU_pres_abspos	-99.618	42.250	0.018
CityWarsaw:CFU_pres_abspos	-364.220	30.186	0.000

Pairwise comparisons of each city pair was carried out by computing linear combinations of fixed effects with the `betta_lincom` function in the `breakaway` package. p-values were generated from a two-sided Wald test, against the null hypothesis that the linear combination of fixed effects is zero.

Table S2. Pairwise comparisons of city population richness estimates

City pair	Estimates	Standard Errors	Lower CIs	Upper CIs	p-values	Adjusted p-value[1]
Athens - Copenhagen	-352.295	77.950	-505.074	-199.516	3.1e-06	0.000
Athens - Rome	54.415	67.698	-78.270	187.100	0.211	0.253
Athens - Warsaw	-392.594	87.263	-563.627	-221.561	3.41e-06	0.000
Copenhagen - Rome	406.710	50.392	307.944	505.476	3.49e-16	0.000
Copenhagen - Warsaw	-40.299	74.641	-186.592	105.994	0.295	0.295
Rome - Warsaw	-447.009	63.859	-572.171	-321.847	1.28e-12	0.000

[1]p-values corrected for multiple comparisons using the Benjamini & Hochberg method

Shannon index

The sample Shannon index was calculated from the normalised data set (n = 10 148 sequences per sample), using the phyloseq package, and a mixed effects model was built with Shannon index as the response variable. City and presence/absence of culturable *Legionella* were specified as fixed effects (including their interaction), and plumbing compartment, nested within building ID, were specified as random effects. Overall significance of the model fixed effects was tested using ANOVA.

*Table S3. Sample Shannon index: ANOVA of mixed effect model. Model formula: Shannon ~ City * CFU_pres_abs + (1 | Bldg_ID / Location_in_building)*

	Sum Sq	Mean Sq	NumDF	DenDF	F value	Pr(>F)
City	6.166	2.055	3	9.991	2.732	0.100
CFU_pres_abs	2.765	2.765	1	135.410	3.674	0.057
City:CFU_pres_abs	2.347	0.782	3	127.919	1.040	0.377

Pairwise comparisons of city Shannon indices were carried out from the model output with the lsmeans package.

Table S4. Pairwise comparisons of city Shannon diversity indices

City pair	estimate	SE	df	t.ratio	p.value[1]
Athens - Rome	0.270	0.389	8.152	0.695	0.896
Athens - Warsaw	-0.699	0.386	7.940	-1.812	0.335
Athens - Copenhagen	-0.559	0.426	10.832	-1.312	0.575
Rome - Warsaw	-0.969	0.383	8.307	-2.529	0.127
Rome - Copenhagen	-0.829	0.424	11.315	-1.957	0.260
Warsaw - Copenhagen	0.140	0.421	11.081	0.332	0.987

[1]p-values corrected for multiple comparisons using the Tukey method

PERMANOVA on distance matrix (adonis)

For beta diversity analyses, we used the compositional Aitchison distance, equivalent to center-log-ratio transformation of the ASV table followed by Euclidean distance calculation. The influence of city and presence/absence of culturable Legionella (plus their interaction) was tested by permutational multivariate analysis of variance (PERMANOVA), using the adonis function in the vegan package.

*Table S5. PERMANOVA of Aitchison distance matrix, computed using adonis function with strata = Building_loc_ID to account for repeated measures. Formula: adonis(clr_dist_matrix ~ City * CFU_pres_abs, strata = Bldg_loc_ID, permutations = 10000)*

	Df	SumsOfSqs	MeanSqs	F.Model	R2	Pr(>F)
City	3	172 134	57 378	14.2983	0.2008	0.0014
CFU_pres_abs	1	22 919	22 919	5.7114	0.0267	0.0005
City:CFU_pres_abs	3	31 990	10 663	2.6573	0.0373	0.0545
Residuals	157	630 027	4 013		0.7351	
Total	164	857 070			1.0000	

Dispersion test (betadispr with permutest)

Multivariate homogeneity of group dispersions was tested for city and *Legionella* pres/abs with the `betadisper` function in the vegan package.

Table S6. Dispersion of groups around centroid, computed using betadispr function in vegan package

Variable		Df	Sum Sq	Mean Sq	F	N.Perm	Pr(>F)
City	Groups	3	12 405	4 135.01	15.3658	10 000	0.0001
	Residuals	161	43 326	269.10			
CFU pres/abs	Groups	1	40	39.89	0.0774	10 000	0.7764
	Residuals	163	84 004	515.36			

Culturable Legionella (CFU/L) by city

To test whether the Legionella culture counts (in CFU/L) differed among the cities, we build a generalised linear model to account for the Poisson distribution of counts. Model fit was improved by addition of random effects, and by separate modelling of zero inflation by city and random effects. Model construction steps are shown in Table S7, final model output in Table S8, and pairwise comparisons of cities in Table S9.

Table S7. Model selection for CFU/L data: GLM with Poisson distribution, with addition of zero inflation and mixed effects

Model name	df	AIC	Counts model terms	Zeros counts model terms
mod.poisson	4	4 497 813	~ City	~ 0 (no zero inflation modelling)
mod.poisson.zi.simple	5	3 097 682	~ City	~ 1 (constant zero modelling)
mod.poisson.zi.city	8	3 097 647	~ City	~ City
mod.poisson.me.zi.simple	7	1 177 111	~ City + (1|Bldg_ID/Location_in_building)	~ 1 (constant zero modelling)
mod.poisson.me.zi.city	10	1 177 104	~ City + (1|Bldg_ID/Location_in_building)	~ City
mod.poisson.me.zi.cityme	12	1 177 090	~ City + (1|Bldg_ID/Location_in_building)	~ City + (1|Bldg_ID/Location_in_building)

Table S8. Culturable Legionella CFU/L by city: zero-inflated mixed effects generalised linear model with Poisson distribution (CFU/L counts and zero counts independently modelled by ~ City + (1|Bldg_ID/Location_in_building)

Model component		Estimate	Std. Error	z value	Pr(>lzl)
Counts	(Intercept)	-3.641	3.311	-1.100	0.271
	CityAthens	6.559	4.159	1.577	0.115
	CityRome	7.610	4.075	1.868	0.062
	CityWarsaw	12.690	4.198	3.023	0.003
Zero counts	(Intercept)	0.608	1.432	0.425	0.671
	CityAthens	-1.839	1.668	-1.102	0.270
	CityRome	0.624	1.697	0.368	0.713
	CityWarsaw	-2.146	1.725	-1.244	0.214

Table S9. Pairwise comparisons of city culturable Legionella counts (CFU/L), computed from GLMM output using the lsmeans package.

City pair	estimate	SE	df	t.ratio	p.value[1]
Copenhagen - Athens	-6.559	4.159	153.000	-1.577	0.395
Copenhagen - Rome	-7.610	4.075	153.000	-1.868	0.246
Copenhagen - Warsaw	-12.690	4.198	153.000	-3.023	0.015
Athens - Rome	-1.051	3.874	153.000	-0.271	0.993
Athens - Warsaw	-6.131	3.669	153.000	-1.671	0.342
Rome - Warsaw	-5.080	3.883	153.000	-1.308	0.559

[1]p-values corrected for multiple comparisons using the Tukey method

Legionella genus in all 16S rRNA data

The proportions of all 16S rRNA sequences that classified as the genus Legionella, and as the species L. pneumophila, were calculated for each sample, as well as the proportion of Legionella genus sequences that were further classified as L. pneumophila. Mixed effects models were built with these proportions as response variables. City and presence/absence of culturable *Legionella* were specified as fixed effects (including their interaction), and plumbing compartment, nested within building ID, were specified as random effects. Overall significance of the model fixed effects was tested using ANOVA.

*Table S10. Proportion Legionella genus and L. pneumophila in 16S rRNA data: ANOVA of mixed effect models. Model formula: Proportion ~ City * CFU_pres_abs + (1 | Bldg_ID / Location_in_building)*

Classification		Sum Sq	Mean Sq	NumDF	DenDF	F value	Pr(>F)
all Legionella genus	City	2.714e-03	9.046e-04	3	9.684	3.396	0.063
	CFU_pres_abs	2.718e-05	2.718e-05	1	107.805	0.102	0.750
	City:CFU_pres_abs	6.271e-05	2.090e-05	3	92.199	0.078	0.972
L. pneumophila only	City	2.446e-04	8.152e-05	3	8.576	0.660	0.598
	CFU_pres_abs	7.710e-06	7.710e-06	1	78.988	0.062	0.803
	City:CFU_pres_abs	1.639e-04	5.464e-05	3	65.954	0.442	0.724

*Table S11. Proportion of all Legionella genus 16S sequences that are further classified as L. pneumophila: ANOVA of mixed effect model. Model formula: L.pneumo proportion ~ City * CFU_pres_abs + (1 | Bldg_ID / Location_in_building)*

	Sum Sq	Mean Sq	NumDF	DenDF	F value	Pr(>F)
City	4 182.162	1 394.054	3	8.032	1.858	0.215
CFU_pres_abs	5 271.805	5 271.805	1	135.533	7.027	0.009
City:CFU_pres_abs	4 761.232	1 587.077	3	115.020	2.116	0.102
